# Transforming organ donation and transplantation: challenges, innovations, and future directions

**DOI:** 10.3389/frtra.2026.1855853

**Published:** 2026-08-05

**Authors:** Rachana Yogesh Patil, Yogesh H. Patil

**Affiliations:** 1 Department of Computer Engineering, Pimpri Chinchwad College of Engineering, Pune, Maharashtra, India; 2 Department of Instrumentation Engineering, D. Y. Patil College of Engineering, Pune, Maharashtra, India

**Keywords:** artificial intelligence, ethical challenges, healthcare systems, organ allocation, organ donation, organ transplantation

## Abstract

Organ donation and transplantation has been one of the most important parts of the modern healthcare system that deals with the end-stage organ failure and enhances quality of life and patient survival. But the existing problems of donor scarcity, lack of efficiency in donors usage, mismatches in different organs, regional imbalance, logistic problems and uncoordinated data sharing system have been still a hindrance in the success of the transplantation systems and it is a critical observation of the present situation in the field of organ donation and transplantation which is justified by data analysis of the tendencies in the usage of the donors, tendencies in organ transplantation and regional differences. The paper also outlines the major challenges in the current systems and the new technologies that will be able to revolutionize the transplantation processes such as Artificial Intelligence, Blockchain, Internet of Medical Things (IoMT) and Big Data Analytics. At the same time, new technologies that would help to extend the “liveness” of organs are also carefully analysed as potential ways to simplify the choices, improve the transparency and logistics. While there are solutions to these technologies, some barriers to the technologies are interoperability, governance, ethical issues and the availability of infrastructure. Lastly, the paper provides the research directions in the future, which revolve around convergence of heterogeneous technologies, development of interoperable data ecosystems and the necessity of having equitable and patient-centered transplantation systems. Overall, this review provides an in-depth opinion of the problems, innovations, and directions that need to be introduced to transform organ donation and transplantation into a more efficient, transparent, and accessible healthcare sector on the international scale.

## 1 Introduction

Organ transplantation has become one of the best and life-saving medical procedures to patients with end-stage organ failure (1). Organ failure remains a significant health burden to the world despite the high level of clinical improvements. The estimates given by the World Health Organization have shown that the number of people that are in need of organ transplantation is way too high compared to the supply that is available and only about 10 percent of the global needs of transplantation are being fulfilled (2). Over 150,000 organ transplants are currently conducted in the world each year but it is still not enough compared to the fast rate of the development of chronic diseases like kidney failure, liver cirrhosis and cardiovascular diseases. Chronic kidney disease is a disease that is alone experienced by about 10 percent of the world population, and most of its patients advance to end-stage renal disease that needs dialysis or transplantation (3).

The mismatch between the demand and availability of organs is also indicated in national transplant systems (4). In the US, the number of patients on transplant waiting lists is more than 100,000 and a new patient is added every 8–10 min. Nevertheless, due to the ongoing campaigns to encourage organ donation, 15–20 patients die every day as they wait to receive transplantation (5). The same problems are noticed in the world, especially in the low- and middle-income nations. In India, as an example, the rate of organ donation is very low in most parts of the country, with most parts having a rate of less than one organ donor per million people, highlighting the shortage in awareness, healthcare facilities and policy execution. The ineffectiveness of the logistics and organ preservation restricts the use of many donated organs, which exposes the systemic gaps that are not related to shortages of donors (6, 7).

**Figure 1:**
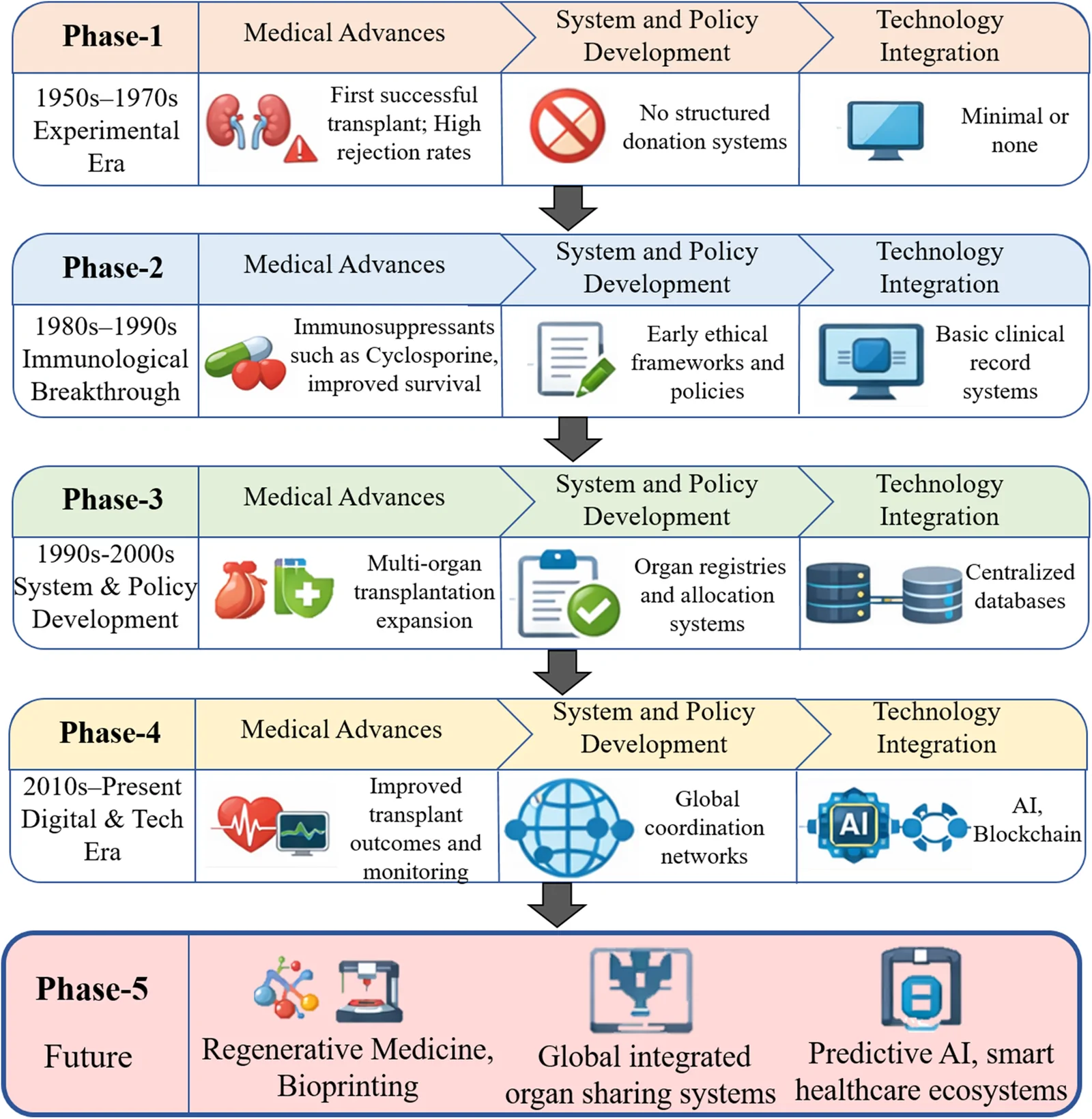
Evolution of organ donation and transplantation systems.

To address such increasing demands, systems of organ donation and transplantation have experienced significant development in the last few decades, shifting towards experimental medical practice to more multidisciplinary, complex systems of healthcare (8). As shown in Figure 1, the initial experimental period (1950s1970s) was marked by poor success because of the high level of immune rejection, poor surgical practices and lack of effective immunosuppressive treatments. Transplantation at this time was more or less experimental with the outcomes being limited by biological and technological factors (9).

The immunological breakthrough era (1980s1990s) was a significant change that took place due to the creation of immunosuppressive drugs like Cyclosporine. These new developments greatly lowered graft rejection and enhanced patient survival making transplantation a viable and more common clinical procedure (10). With the increase in success rates, there was a rapid increase in the demand of transplantation, which increased the mismatch between supply and demand of organs.

The next step in the early 2000s was devoted to the development of the system and the policy, which is characterized by the creation of the organized organ donation systems, such as national registries, allocation systems, and the regulations (11). These systems were meant to make sure that there was fairness, transparency and efficiency in the organ distribution and also encourage the people to be involved in the donation programs. However, with the growing number of transplantation systems, several ethical and operational problems have emerged such as models of consent, fairness of allocation, and cross-border organ sharing (12).

In recent years organ transplantation has entered a new digital and technological era where increasingly complex computer and biomedical technologies are being introduced. AI is being applied to enhance the matching of donors and recipients and predict the success of the transplant, whereas technologies such as blockchain are under consideration to increase the transparency, traceability, and security of data in organ allocation. The development of organ preservation methods, including machine perfusion and the development of cold-chain logistics have also contributed to the extension of organ viability and minimization of wastage. Nevertheless, their complete clinical integration is hindered by such issues as data privacy, algorithmic bias, interoperability, and uneven uptake of technology (13).

In the future, transplantation is heading in the direction of more intelligent and regenerative. Bio printing, stem cell treatments, and predictive analytics are some of the technologies that will help to decrease the use of donor organs and enhance long term results. Concurrently, the international organ-sharing platforms and reliable data-sharing networks can increase the level of coordination and equity in distribution (14). However, such developments have significant ethical, regulatory, and social issues that should be taken into consideration to implement it in a responsible and fair manner.

Even after decades of development, the transplantation systems are still disjointed, and the infrastructure, alignment of policies, and integration of technologies are still missing. It is evident that a further analysis of current practice, new innovations, future trends, in particular how medico legal healthcare system and technological innovations can be combined in order to address the current constraints, is required (15).

Despite considerable advances in organ transplantation research, existing review articles have predominantly focused on individual aspects such as artificial intelligence, blockchain, organ preservation, or digital health technologies in isolation. A comprehensive synthesis that integrates these emerging technologies with global transplantation trends, donor utilization patterns, implementation challenges, and future clinical translation remains limited. To address this gap, the present review not only summarizes current advances but also critically compares the strengths, limitations, clinical readiness, and translational potential of these technologies within a unified transplantation framework. By combining technological advances with clinical and operational challenges, this review offers an integrated framework to guide future research and support the development of more efficient, secure, and patient-centered transplantation systems.

## 2 Review methodology

**Figure 2:**
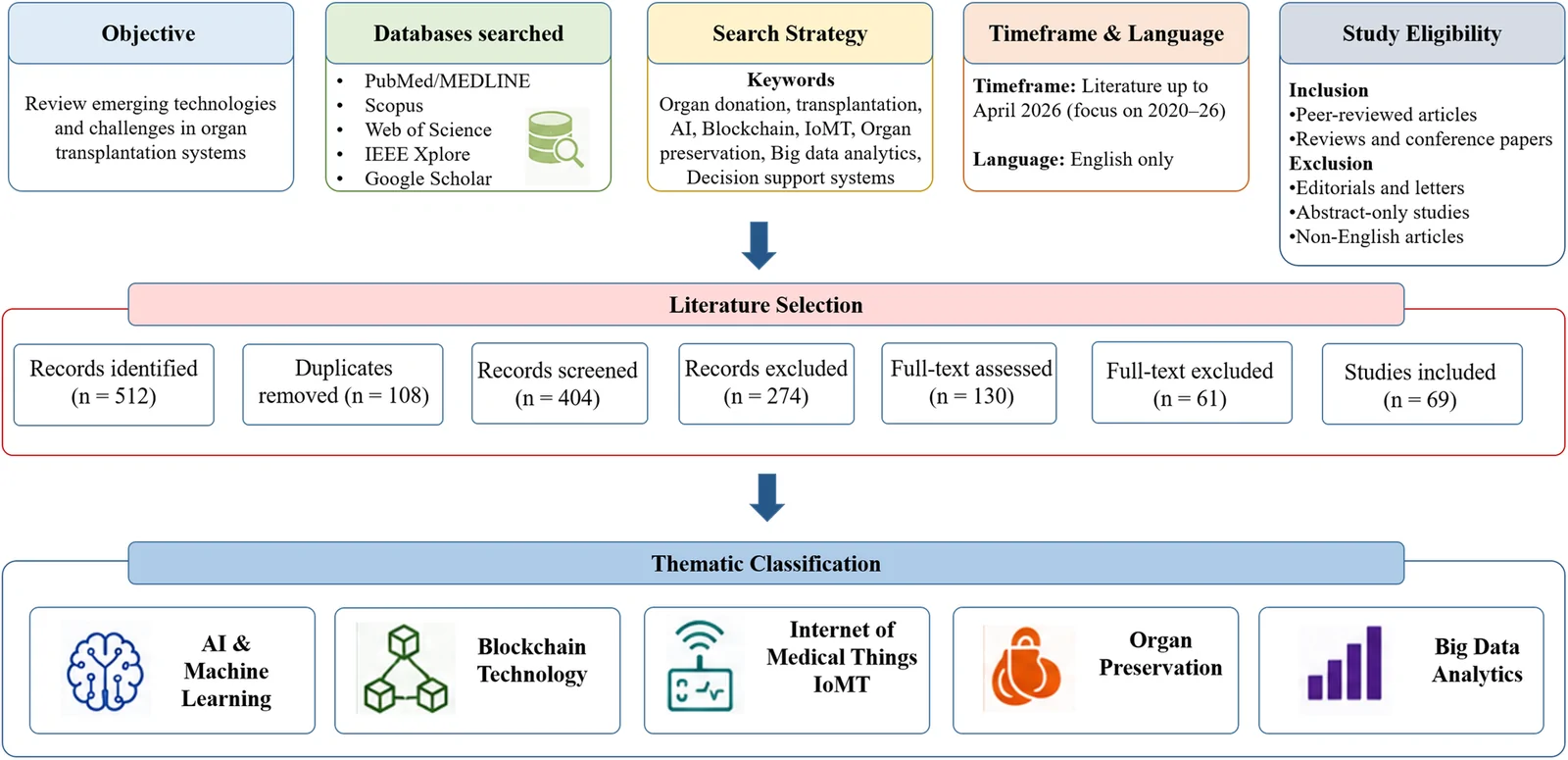
Review methodology and study selection process.

This study was a narrative review, which involved conducting a structured literature search to identify and collate evidence related to emerging technologies and problems of organ donation and transplantation systems. The methodology used for the overall review is shown in Figure 2.

Five major electronic databases (PubMed/MEDLINE, Scopus, Web of Science, IEEE Xplore and Google Scholar) were used for a comprehensive literature search. The search strategy used included the use of various combinations of keywords for organ donation and organ transplantation as well as emerging technologies such as:

The terms used in the context of this project are “organ donation,” “organ transplantation,” “artificial intelligence,” “machine learning,” “blockchain,” “Internet of Medical Things (IoMT),” “organ preservation,” “big data analytics,” and “decision support systems”.

The literature search included publications up to April 2026, with a special focus on those published in the period from 2020 to 2026, and also seminal publications from previous years where they were relevant to provide historical and technological background. English language publications only were taken into account.

Relevant conference papers, review papers and peer-reviewed journal papers on organ transplantation systems and emerging technologies were included. Editorials, letters, abstract-only publications, non-English articles, duplicate studies and studies that were not directly relevant to the scope of this review were excluded.

The first search resulted in 512 records as shown in Figure 2. 108 duplicate studies were excluded and 404 studies were screened for title and abstract. 274 articles were then excluded and 130 full text articles were evaluated for eligibility. After full-text evaluation, 61 studies were discarded based on the pre-established criteria and 69 studies were included in the final qualitative synthesis.

The studies included were then grouped into five thematic areas, for a structured and focused analysis:

1. Artificial Intelligence and Machine Learning;
2. Blockchain Technology;
3. Internet of Medical Things (IoMT);
4. Advanced Organ Preservation Technologies; and
5. Big Data Analytics and Decision Support Systems.

The literature selected was critically synthesized to highlight the latest developments, implementation barriers, research gaps and future prospects for the development of next generation transplantation systems.

## 3 Current landscape of organ donation and transplantation

The landscape of organ donation and transplantation has evolved into a multi-dimensional and multi-faceted world with medical, organizational and regulatory components. Although there has been great development in surgical procedures and after-transplant management, there is still an unequal distribution of donor organs in different regions (16). The current transplantation systems are structured and shaped by the variations in health care systems, legal, cultural and technological orientations and adoption, resulting in large disparities in access and outcomes worldwide.

### 3.1 Global distribution of solid organ transplantation

**Figure 3:**
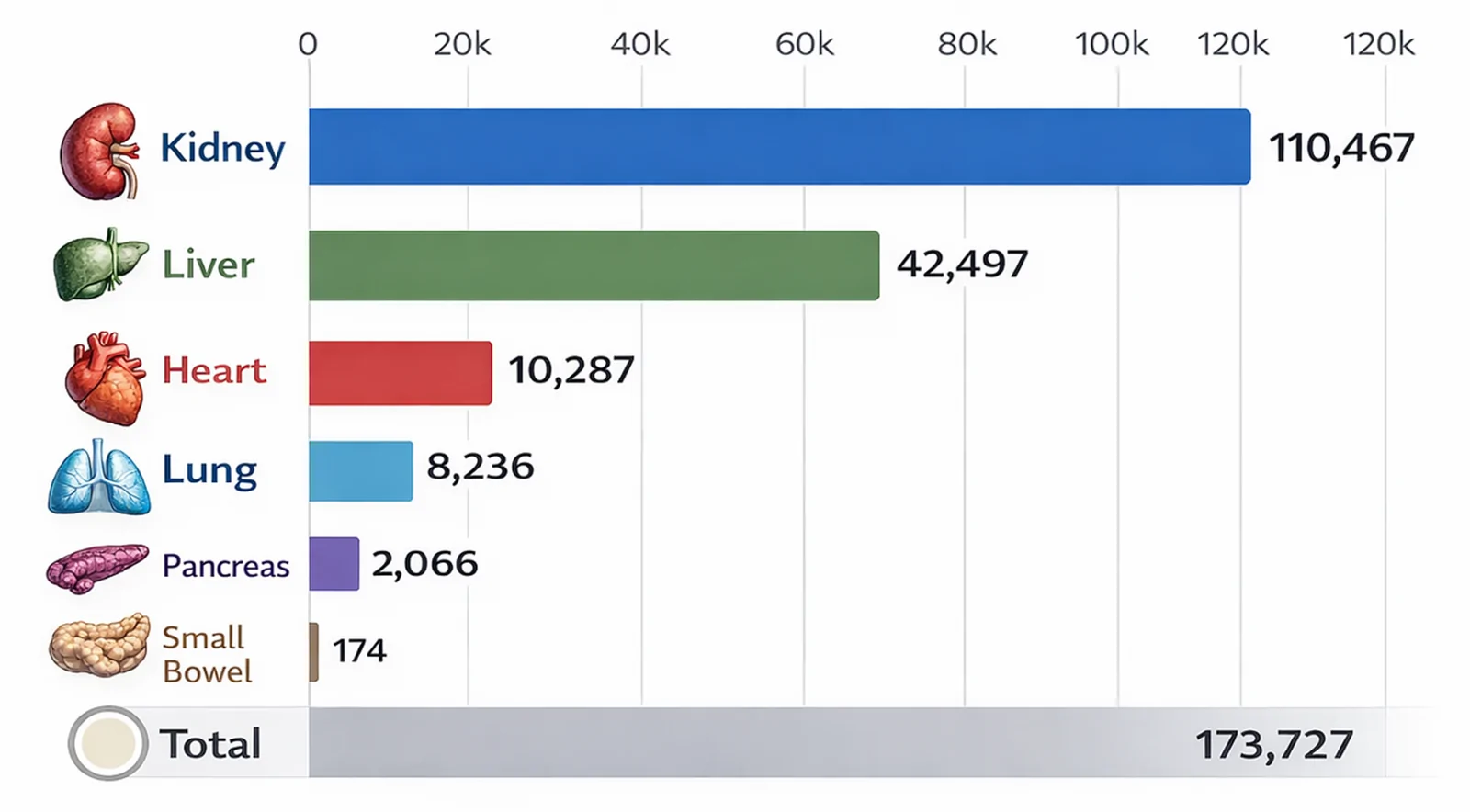
Global distribution of solid organ transplants by organ type (data derived and visualized from the global observatory on donation and transplantation 2025) (18).

In 2024, as per Global Observatory on Donation and Transplantation, 173,727 solid organ transplants were reported in 92 countries around the world. Figure 3 demonstrates that most of the procedures are kidney transplantation, with 110,467 transplants (≈64%), largely due to the high incidence of chronic kidney disease and the possibility of living and deceased donation.

The second largest share is liver transplantation, comprising 42,497 procedures (≈24%), which is a consequence of increasing the load on liver diseases. Heart and lung transplants have smaller proportions, 10,287 (≈6%), and 8,236 (≈5%) cases, respectively, as they are more complex and need more resources (17).

Transplantation of the pancreas (2,066; ≈1) and small bowel (174; <0.1) is not common, in part because of clinical difficulties and limited indications. In general, the distribution shows the prevalence of kidney and liver transplantation, whereas other types of organs are not used as actively.

Although pancreas and intestinal transplantation account for a relatively small proportion of global transplantation activity, these procedures remain clinically important for carefully selected patient populations. Pancreas transplantation offers significant benefits for selected patients with insulin-dependent diabetes mellitus, while intestinal transplantation represents a life-saving intervention for patients with irreversible intestinal failure. Their comparatively low procedural volumes largely reflect the complexity of recipient selection, donor availability, and the highly specialized expertise required for these procedures.

### 3.2 Donor utilization efficiency

The efficiency of donor utilization is an essential performance indicator of transplantation systems that indicates the capacity to transform identified donors into successfully used organ donors (18). The utilization rates were calculated in this research on overall deceased donors (DD), donation after brain death (DBD), and donation after circulatory death (DCD) during the years 2014–2024. The utilization rates are defined as follows:

$$\mathrm{DD}\;\mathrm{Utilization}\;\mathrm{Rate}=\frac{\mathrm{Total}\;\mathrm{UtilizedDD}}{\mathrm{Total}\;\mathrm{Actual}\;\mathrm{DD}}*100$$

$$\mathrm{DBD}\;\mathrm{Utilization}\;\mathrm{Rate}=\frac{\mathrm{Total}\;\mathrm{Utilized}\;\mathrm{DBD}}{\mathrm{Total}\;\mathrm{Actual}\;\mathrm{DBD}}*100$$

$$\mathrm{DCD}\;\mathrm{Utilization}\;\mathrm{Rate}=\frac{\mathrm{Total}\;\mathrm{Utilized}\;\mathrm{DCD}}{\mathrm{Total}\;\mathrm{Actual}\;\mathrm{DCD}}*100$$

**Figure 4:**
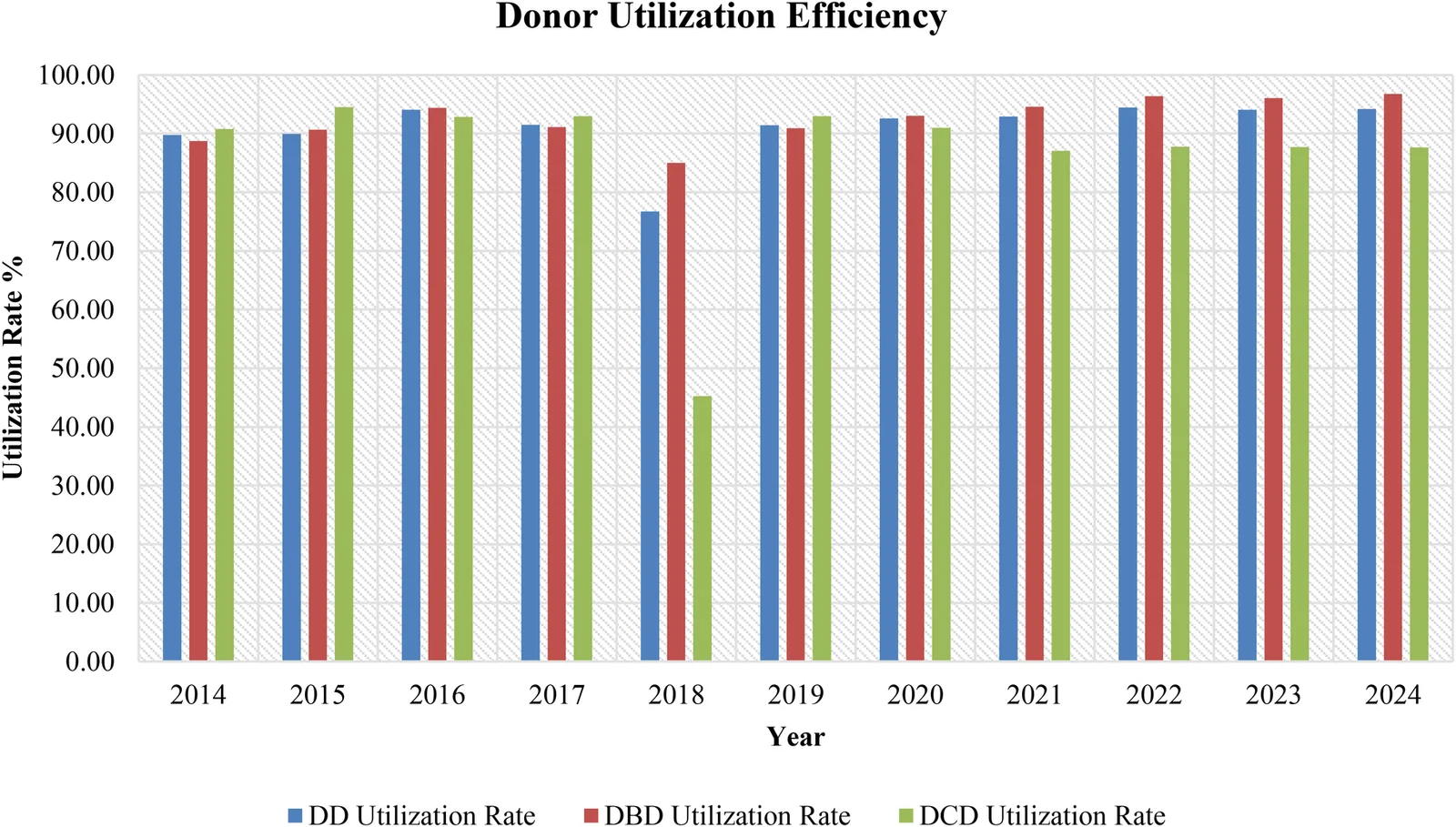
Donor utilization efficiency analysis (2014–2024) (data derived and visualized from the global observatory on donation and transplantation 2025) (18).

Figure 4 shows how the efficiency of donor utilization has changed with time during the study period. In general, the DD utilization rate shows a rather steady and high level of performance, with the figures ranging between 89.77% in 2014 and 94.20% in 2024, which implies slow but steady changes in organ recovery and allocation practices.

Another interesting fact is that the utilization of DBD is always high, with the percentage increasing to 96.76 in 2024, compared to 88.74 in 2014, so it is the most efficient donor type. This tendency indicates the existence of well-developed clinical guidelines, enhanced organ viability, and simplified decision-making procedures related to brain-death donors.

Conversely, there is a high time variability in the use of DCD. Although the utilization rates are relatively high in 2014–2017, it is clear that they dropped to 45.23% in 2018, and then recovered and stabilized at about 87.88 percent in recent years. This variability indicates the real-life issues of DCD processes, including the necessity to retrieve organs quickly, short preservation period, and more stringent eligibility requirements.

The second valuable lesson is the consistent difference in the rates of DBD and DCD use. The gap between DBD and DCD decreases in some years, but the use of DCD is still significantly lower than that of DBD, reflecting structural and operational inefficiency peculiar to circulatory deaths.

An interesting exception is observed in 2018, where a sharp decline in utilization rates, particularly among DCD donors, is evident. The underlying reasons for this decrease cannot be determined from the aggregated data analyzed in this study. This observed variation could be due to changes in reporting practice, donor selection criteria, allocation policies, etc. Further research into this decline is required at a more detailed level, along with detailed data at the registry level, to identify factors affecting the decline.

Overall, the findings suggest that the application of deceased donors has become relatively “best” but DCD application is a field which could be developed further. Refining clinical procedures, developing better preservation techniques and mechanisms of coordination may result in significant expansion of the pool of organs available for transplant, thus helping to reduce organ shortages, if DCD is used more widely.

### 3.3 Organ-wise transplantation patterns and donor source distribution-global

Organ-based transplantation activity data was analysed from all available data 2014–2024 to give a complete picture of the transplantation activity. Major organs analyzed are kidney, liver, heart, lung, pancreas and small bowel with corresponding donor sources.

**Figure 5:**
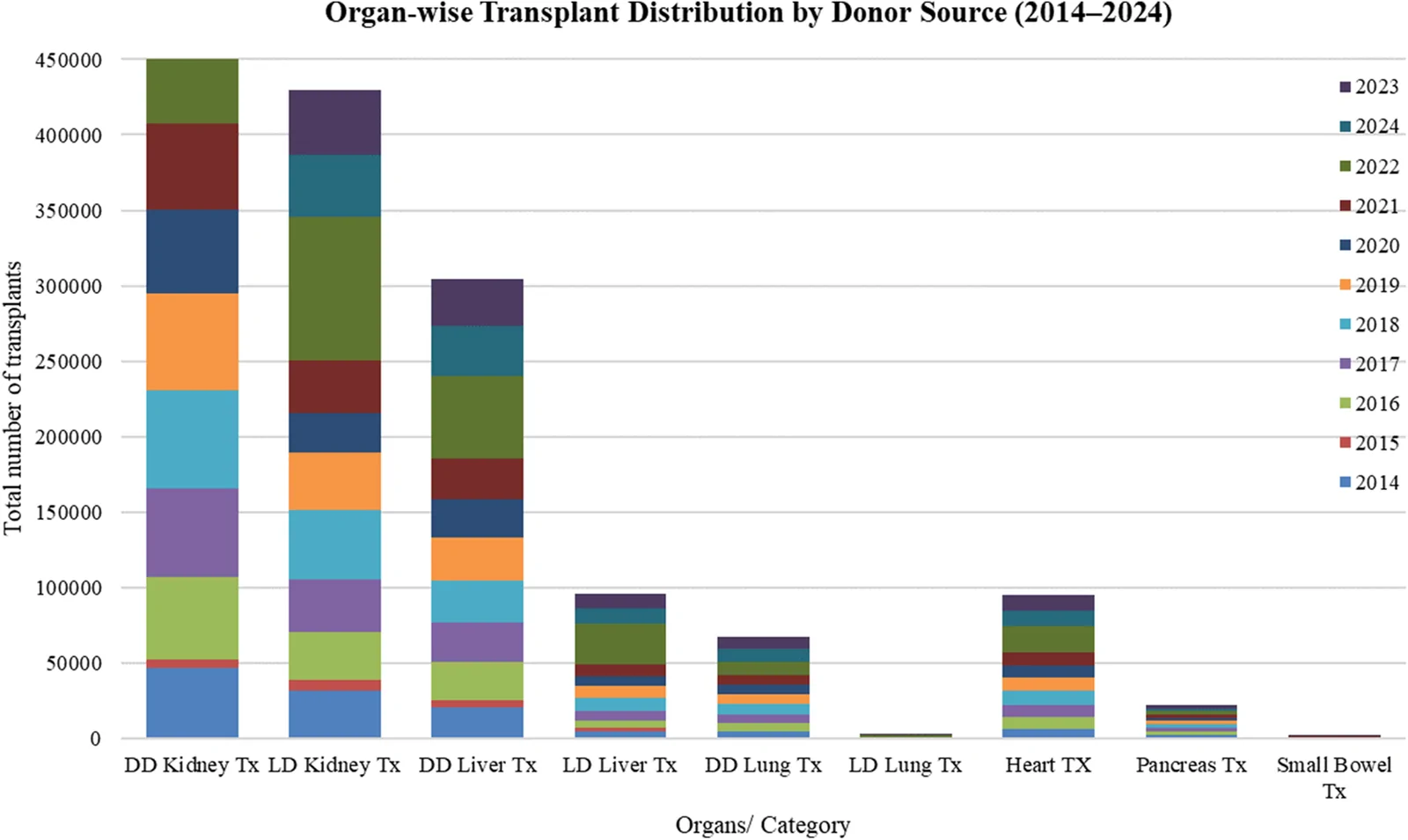
Comparative analysis of organ transplants by donor source across Major organs globally (2014–2024) (data derived and visualized from the global observatory on donation and transplantation 2025) (18).

Kidney transplantation is the largest share of all transplants, accounting for 40% of the total activity during the study period as displayed in Figure 5. In 2024, 110,467 kidney transplants were carried out of 69,472 deceased donors (DD) and 40,995 of living donors (LD). Over the years the trend is the same—in 2018, over 111,000 kidney transplants were registered, of which 65,491 were DD and 46,082 were LD. High good attendance in living donor transplant is a sign of scalability and maturity of kidney transplant programmes (19).

The second is the liver transplantation according to the mixed donor model. In 2024, 42,483 liver transplants were performed, including 32,618 DD and 9,865 LD cases. However, the number of living donors is less than that of kidney transplants, suggesting some degree of diversification of the donor sources.

Conversely, thoracic transplants, particularly heart and lung, are virtually reliant on dead donors. In 2024, 10, 287 heart and 8,236 lung transplants were done with minimal or no input of living donors. This is a manifestation of natural clinical constraints and dependence on donor availability of the dead. Transplants of the pancreas and small bowel are still considered rather rare, with 2,066 and 174 performed respectively in 2024. Their low rates are always a sign of more complexity and lower applicability than kidney and liver transplants.

All in all, the activity of transplants does not show even distribution among the types of organs, with the most prevalent being kidney and liver transplants. Depending on living donors, especially to provide kidney (∼37%) and liver (∼23%), assists in compensating the shortage of deceased donors, but also creates the issue of safety and sustainability of the donor. A notable increase in transplant activity is observed in 2022, particularly for kidney and liver transplantation. However, the factors underlying this increase cannot be determined from the aggregated data analyzed in this study. The observed trend may reflect recovery from prior disruptions, changes in reporting practices, or improvements in transplantation activity, but further investigation using detailed registry data is required.

To conclude, although there are successful transplant pathways that have become scale and efficiency-driven, there are still ones that are limited due to clinical, logistical, and infrastructural constraints. To develop a more balanced and efficient transplantation ecosystem, it is important to address these gaps by means of better donor management, more advanced preservation technologies and better allocation strategies.

### 3.4 Regional distribution of transplantation rates

To facilitate comparisons between regions, 2024 transplant activity was normalized per million population (pmp) by major organs. The transplantation rate is defined as:

$$\mathrm{Transplant}\;\mathrm{Rate}(\mathrm{pmp})=\frac{\mathrm{Total}\;\mathrm{Transplants}}{\mathrm{Population}}*10^{6}$$

**Figure 6:**
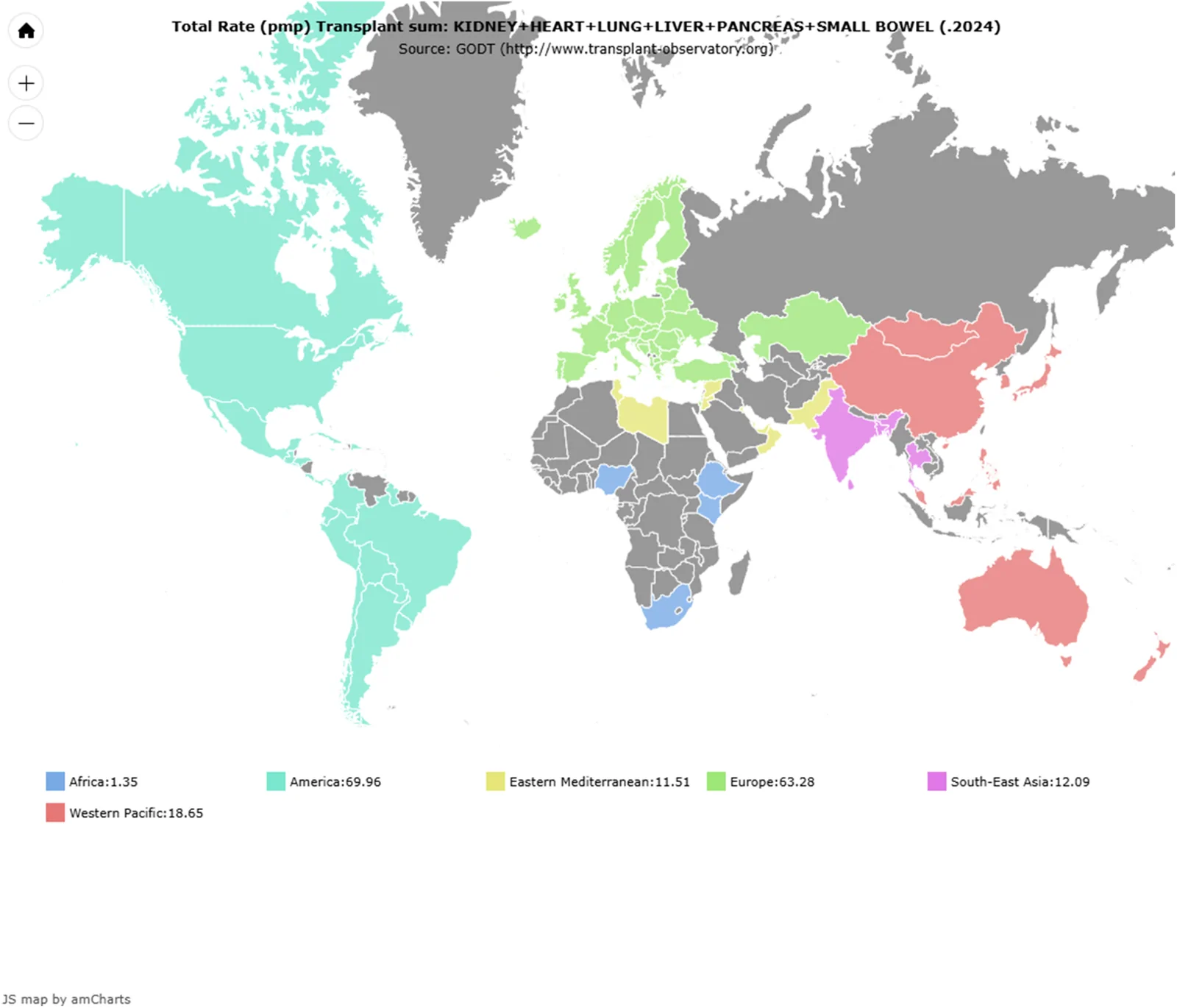
Regional distribution of transplantation rates (data derived and visualized from the global observatory on donation and transplantation 2025) (18).

As can be seen from Figure 6 there are definite differences in the world. The leaders are the Americas (69.96 pmp) and Europe (63.28 pmp) which shows that the system of transplants, clinical care, and organ distribution is good. On the other side, South-East Asia (12.09 pmp) and Eastern Mediterranean (11.51 pmp) are quite low, indicating the restriction of the donor awareness, healthcare facilities and coordination. The rate of Western Pacific is moderately active (18.65 pmp) and presupposes partial development, the lowest is Africa (1.35 pmp) which is a reason of serious concerns with access, infrastructure and resources.

Overall, the activity of transplants is uneven across the regions, and is also influenced by the capacity and technological development of healthcare, policy framework and socio-economic factors (20). Good donor programs, advanced preservation and integrated data systems, and barriers along the transplant pipeline, characterize high performing areas. The results suggest that some interventions are required to achieve a reduction in the disparities between the regions and to improve access to transplantation globally, such as policy standardization, and introduction of technology.

## 4 Limitations and challenges in current systems

**Figure 7:**
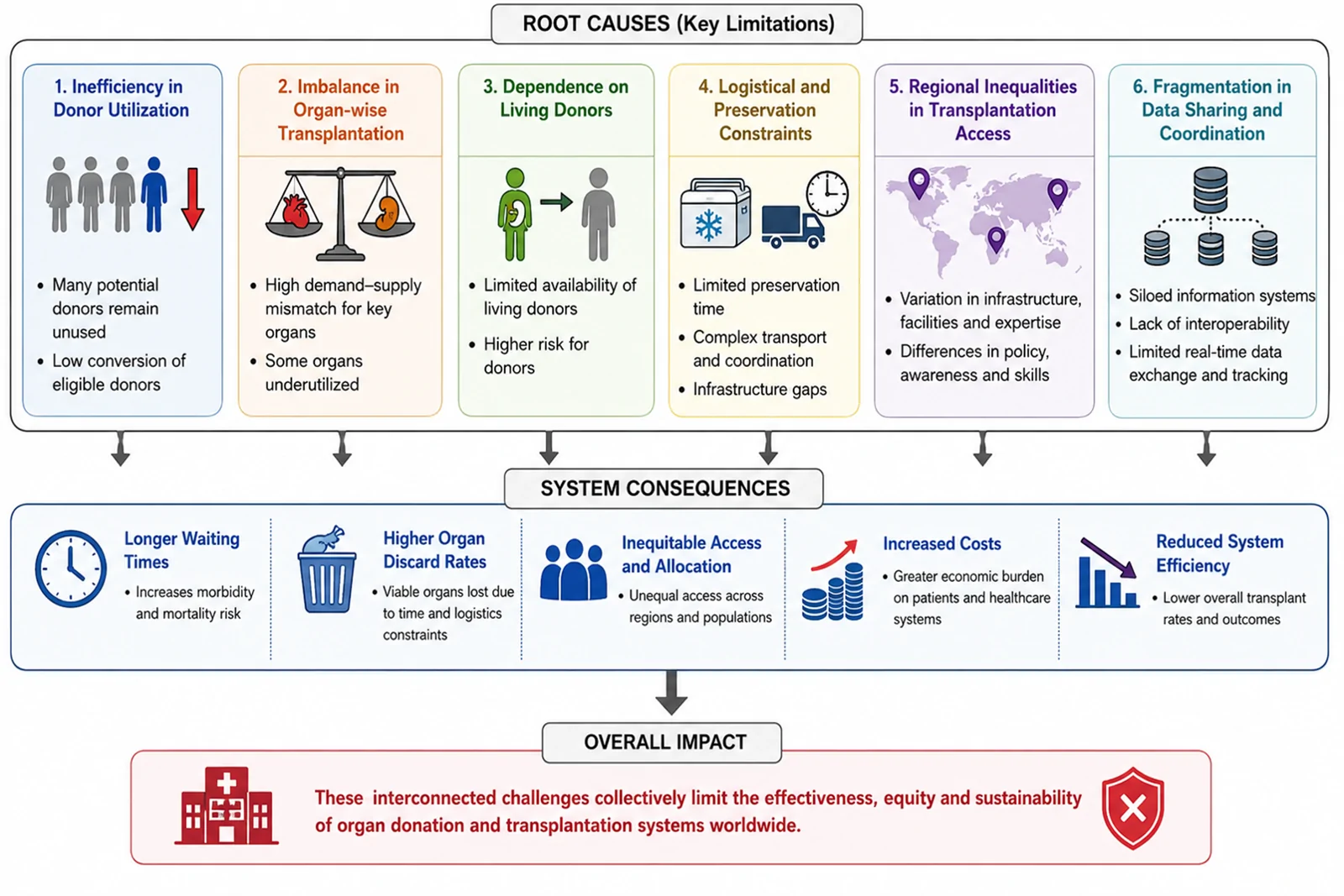
Key limitations and challenges in organ donation and transplantation systems.

Although there is constant development in the field of organ donation and transplantation, the existing ecosystem is still limited by a number of critical constraints which influence the efficiency, equity, and scalability. The discussions above show that the issues are systemic and relate to the use of donors, organ allocation, infrastructure, and data management. All these restrictions hamper the capacity of transplantation systems to satisfy the increasing demand across the world. These issues are clinical, operational, and systemic in nature and influence different phases of the transplantation process. According to Figure 7, donor utilization gaps, imbalance in organ-specific transplantation, use of living donors, regional inequality, logistical limitations, and data sharing fragmentation are some of the inefficiencies. All these challenges are addressed in the following subsections.

### 4.1 Inefficiency in donor utilization

The efficiency of the donor utilization is a major issue in the transplantation system since there is still a big gap between the number of donors identified (actual) and those whose organs are actually used in transplantation. This ineffectiveness directly restricts the effective supply of organs, even with the growing work on the registration and awareness of the donors. The difference means that there are losses at several points of the transplantation chain, such as the social awareness and assessment of the donor, organ recovery, preservation, and distribution (21).

One of the most important findings of the analysis is the difference in the efficiency of utilization between donation after brain death (DBD) and donation after circulatory death (DCD). Utilization rates are always high as DBD donors have constant organ perfusion prior to retrieval and therefore keep the organs viable. On the other hand, DCD donors are more likely to have warm ischemia, an event following circulatory arrest which significantly affects organ quality. Consequently, the use of DCD has more variable rates with lower rates, reflecting clinical constraints.

There are other reasons for underutilization of donors other than physiological ones, such as logistical and operational issues. Various stakeholders like hospitals, organ procurement organizations and transplant centers have to work seamlessly for the process of transplantation. Any delay in any of the communication, decision making or transport process can result in organ discard, especially due to the short timeframe of successful organ transplantation (21).

Also, strict donor selection criteria are contributing to restrict the usage. Even the organs with only marginal suitability for transplantation are generally rejected on account of fear of what might happen to it after transplantation. This may further be complicated if there is no advanced predictive tool that can be used to assist in clinical decision making.

Overall, this inefficiency in donors use is due to a series of clinical, logistical and fragmented coordination mechanisms. This challenge should be addressed with the aim of maximizing the number of donor organs and improve the transplantation programmes in general.

However, great progress has been made to make DCD transplantation more possible. The use of normothermic regional perfusion (NRP) and ex situ machine perfusion technologies have facilitated the assessment of organs, minimised ischemic injury and enhanced the use of organs from DCD donors. These have been successful in some instances, especially in liver and kidney transplantation, and have helped to grow the number of DCD programs in certain nations. However, there are many challenges to widespread adoption, including resource needs, technical knowledge, and regulatory and ethical differences.

### 4.2 Imbalance in organ-wise transplantation

High organ-specific imbalance of transplantation activity is the current issue of transplantation. As outlined in Section 2, the number of organs transplanted is highly skewed with a small number of kidneys and liver, and a significantly smaller number of other organs, including heart, lung, pancreas and small bowel. This imbalance is the result of the clinical feasibility, donor availability and available infrastructure for the various transplant programs.

Kidney transplantation has dominated the world because of various reasons such as the fact that end-stage renal disease is relatively common, the surgery is relatively standardized, and the donor is either deceased or alive (22). On the same note, liver transplantation enjoys the advantage of a partially diversified pool of donors, which include deceased donors, living donors and in some instances split or domino donors. These features allow increased transplant volumes and more scalable programs.

Transplants of thoracic organs, such as the heart and lungs are, on the contrary, highly restricted. These transplants are completely reliant on deceased donors and require strict physiological compatibility and very short time availability, which limits the appropriateness of donors. Surgical operations are also less common and this is further aggravated by the nature of surgical procedures and the special infrastructure required (23). This results in a slight decrease in heart and lung transplantation compared to kidney and liver. Lower activity is seen in pancreas and small bowel transplant cases, which is mainly due to the high surgical risk, stringent recipient selection criteria and chronic indications for clinical use. They have a low role in the total transplantation activity because of the following reasons:

This asymmetry means that there is a unequal distribution of resources and progress in the types of organs. This results in less or more complicated transplants, fewer access and wait times for patients (24). To correct this imbalance, dedicated investments in infrastructure, clinical practice and the introduction of a more appropriate strategy to engage donors to make the transplantation system more balanced and equal.

### 4.3 Dependence on living donors

One of the issues that have been experienced in the modern transplantation systems is the overreliance on living donors especially in kidney and liver transplantation. Even though living donation has significantly contributed to increasing the number of transplants and reducing the wait time, there are also some severe ethical, medical, and sustainability concerns. In some circumstances, it can be substantial, due to a lack of deceased donors and/or procurement systems. Nonetheless, living donation has been also shown to be a viable and clinically successful transplantation strategy particularly in kidney transplantation (25). The example of kidney transplantation, in particular, is a good illustration that it is highly dependent on the living donors, as the operation is rather safe and the donors can still live with one kidney.

Similarly, in liver transplantation, the living donor has gained popularity due to regenerative capability of liver, which enables half an organ to be donated. All these issues have predisposed living donation as an option and a frequent necessity to overcome organ shortages.

But this reliance brings up serious ethical issues. Living donors are healthy people that go through major surgical operations with no direct medical advantage, subjecting them to possible surgical risks, long-term health effects, and psychological pressure. To guarantee the safety of donors, informed consent, and aftercare is one of the main issues related to transplantation practice.

living donors also points to the failure in the identification of deceased donors, consent, and efficiency of utilization. Ideally, an effective transplantation system must be in a position to satisfy a considerable percentage of the organ demand by deceased donation and hence reduce the risks posed to the living persons (26). The fact that the donor programs are still reliant on living donors implies that the deceased donor programs are not well-optimized.

Moreover, the differences in the availability of living donors may cause inequities in transplantation opportunities because not all patients have appropriate or willing donors. It may result in the disparity in access to transplantation services depending on social, family, or economic backgrounds.

In conclusion, although the undeniable importance of living donation in the sustainability of the transplantation systems, the excessive use of the latter highlights the need to reinforce and make more efficient the programs of deceased donors, as well as to create more balanced and sustainable organ distribution systems.

### 4.4 Regional inequalities in transplantation access

One of the most important problems of the organ donation and transplantation systems is the regional differences, which has a significant impact on the equitable distribution of life-saving procedures. The region-by-region differences in transplantation rates (population normalized) reveal that there are significant acute discrepancies in the system's performance and capacity to provide healthcare.

The high performance areas (Americas and Europe) are where the rates of transplantation are much higher, reflecting the high levels of development of healthcare systems, procurement systems and good policy frameworks that encourage donation and allocation. The higher level of awareness of the population, the clinical expertise, and access to the latest technologies to preserve and transplant organs are also beneficial to these areas (27).

Conversely, other areas like Africa, South-East Asia and some of the Eastern Mediterranean have much lower rates of transplantation. Such differences are usually fuelled by the inadequacy of healthcare facilities, the absence of awareness programs by donors, and the absence of uniform policies regarding organ donation. The cultural, social and religious factors might also affect the rates of donor consent in most of the low performing areas, which further limits the supply of organs.

Economic limitations are very important in influencing these inequalities (28). Poor funding of transplantation programs, insufficient training of specialized medical staff, and unavailability of sophisticated medical technologies are some of the factors that reduce the transplantation activity. Also, the differences in the data reporting and registry systems make it difficult to monitor and evaluate the performance of transplantation at the regional level.

These disparities are not merely pointers to disparities in healthcare capacity, but also highlight other issues of health equity in the world. The patients in low performing areas are likely to receive long lines or no access to transplants that lead to preventable disease and death.

The only way to address the issue of the discrepancy of the region is to act on the national and international level, i.e., to strengthen the healthcare infrastructure, to raise the awareness of people, to equalize the policies and to enhance the collaboration between the regions. These gaps need to be bridged so as to make transplantation services available to everyone in the world.

### 4.5 Logistical and organ preservation constraints

Organ transplantation is a time-sensitive process by definition, and the logistical organization and organ preservation are the key factors that define the success of the process. Although there have been improvements in the surgical procedures, transportation, coordination and preservation inefficiencies still restrict the successful use of the donor organs.

The limited viability window of organs is one of the major challenges (29). The cold ischemia time of each organ type is very short, and after it, the probability of the graft failure grows considerably. An example of this is that the heart and lungs should be transplanted within a few hours, but the kidneys have a longer shelf life. Such limitations put a huge burden on the coordination among donor-receiver, transport systems, and transplant centers.

The delays in retrieval, transportation and distribution may lead to lost transplant opportunities. It is a process that has several stakeholders working in various locations and sometimes the decision-making process has to be made in real-time and within a very limited time frame (30). Any failure of communication or coordination may lead to discarding of organs especially in geographically far-flung areas.

This becomes even more complicated when it comes to the case of donation after circulatory death (DCD) where organs are more vulnerable to ischemic damage since there is no blood flow to the organs before retrieval. This heightens the need to preserve and transport it quickly, and thus DCD transplantation is strongly reliant on effective logistics and modern preservation methods.

Also, the restrictions in organ preservation technologies help to decrease the utilization rates. Although the conventional cold storage procedures are highly applicable, they might not be effective in preserving the quality of organs in the long run (31). New technologies like machine perfusion have better preservation and are not as yet universal because of the cost and infrastructure needs.

In general, there are logistical and preservation issues to consider that can cause the transplantation process to have bottlenecks, not only in the number of organs available, but also in their quality. Thus, better coordinated systems, investments in advanced preservation technologies and implementation of integrated real time monitoring and transportation systems are needed to overcome these barriers and enhance transplantation efficiency.

## 5 Emerging innovations in organ donation and transplantation

The field of organ donation and transplantation has seen remarkable changes with the advent of digital technologies, data-driven methods and smart systems. These innovations are designed to overcome some of the drawbacks of the current systems to match donors and recipients better, provide real-time monitoring, and facilitate secure and transparent data sharing (32). The section focuses on some of the major technological advancements that are transforming the transplantation process and improving the overall effectiveness of transplantation systems.

### 5.1 Artificial intelligence (AI) in organ matching and prediction

AI has become one of the most researched and promising technological developments in organ transplantation, especially in overcoming the problem of donor recipient matching, organ use, and prediction of outcomes. In contrast to the conventional rule-based allocation systems, AI-based methods use extensive clinical data to simulate multivariate and complicated associations between the attributes of donors, recipient features, and transplant outcomes.

The existing literature suggests that AI applications in the field of transplantation can be divided into four large domains: donor-recipient matching, organ offer and discard prediction, post-transplant outcome prediction, and policy simulation to optimize allocation. Among them, the donor–recipient matching and allocation decision support are some of the most practically relevant use cases as they directly affect real-time clinical decision-making.

One of the main lessons of the current literature is that AI is not always more effective than the traditional statistical models. Rather, it depends on its context-specific effectiveness with significant improvements in its effectiveness noted when the models incorporate donor, recipient and process level variables. As an example, externally validated models like D-TOP have been found to have better predictive performance than traditional indices, and other studies have found only a marginal improvement over regression-based methods. This highlights the operational efficiency gains of AI's usefulness, beyond just predictive accuracy, and its role in decision-making.

As shown in Table 1, the field of organ transplantation has seen significant advancements of AI applications in various areas such as donor-recipient matching, organ discard prediction, post-transplant outcome prediction, and optimization of allocation policies. While the predictive capabilities are encouraging, most of the studies are retrospective and use registry-based or single-center data. Another common finding in the literature is the lack of external and prospective validation, which can introduce doubts about the generalizability of the models in other patient populations and healthcare environments. In addition, there are several studies that identify interpretability, data drift and integration into the current transplantation workflow as challenges. Interestingly, there are some areas of AI application that have relatively greater potential for translation, such as donor matching and discard prediction, while fairness optimization and policy simulation are largely experimental. The findings indicate a need for further studies that go beyond algorithm development and include prospective multicenter validation, explainable AI approaches, and incorporation into clinical decision-making procedures to promote responsible use of these algorithms in transplantation practice. Overall, the current evidence suggests that AI should be viewed as a decision-support technology that augments, rather than replaces, clinician judgment and existing allocation systems.

**Table 1:** Comparative analysis of AI-based approaches in organ transplantation.

Study	Task & organ	Model/data	Key outcome	Insight	Major limitation
Berrevoets et al. (33)	Allocation policy	Counterfactual survival + queueing	Improved life-years (simulation)	Strong for policy modeling	Simulation-only evaluation
Anagnostides et al. (34)	Heart allocation	Dynamic optimization (UNOS)	Better allocation efficiency	System-level impact/strong policy modeling	No clinical implementation
Alowidi et al. (35)	Kidney matching	ML classifiers + ranking	High matching accuracy (∼98%)	Prototype-level tool/clinically usable	No external validation
Ali et al. (36)	Kidney allocation	Deep survival models	Better than KDPI	Clinically relevant/strong policy modeling	Retrospective study design
Ashiku et al. (37)	Kidney discard prediction	Deep learning	∼10% improvement	Practical utility Clinically relevant	Dataset bias; no prospective testing
Yoo et al. (38)	Kidney survival prediction	Random forest, survival models	C-index ∼0.80	Interpretable	Requires contemporary validation
Bae et al. (39)	Kidney outcomes	ML vs regression	Similar performance	Challenges ML superiority	Limited ML advantage
Lau et al. (40)	Liver graft prediction	Random forest	AUC ∼0.82	Strong benchmark comparison	Limited generalizability
Börner et al. (41)	Liver matching	Deep learning	High accuracy (∼95%)	Limited generalizability	Limited interpretability
Miller et al. (42)	Heart prediction	ML under temporal shift	Performance drop	Highlights data drift	Vulnerable to data drift
Zafar et al. (43)	Lung matching	Registry + web tool	C-stat ∼0.67	Clinically usable	Moderate predictive performance
Sharma et al. (44)	Lung prediction	Interpretable ML	Moderate accuracy	High usability	Early-stage methodology
Ding et al. (45)	Liver fairness	Debiasing ML	Improved fairness	Early-stage work	Simulation-only evaluation

### 5.2 Blockchain for secure and transparent data management

The blockchain technology has become one of the potential solutions to solve the key issues associated with data fragmentation, absence of transparency, and inter-organizational trust in the systems of organ donation and transplantation. Blockchain is a decentralized, tamper-resistant, and auditable method of sharing data safely among stakeholders, such as hospitals, procurement organizations, transplant centers, and regulators, unlike centralized databases (46).

**Figure 8:**
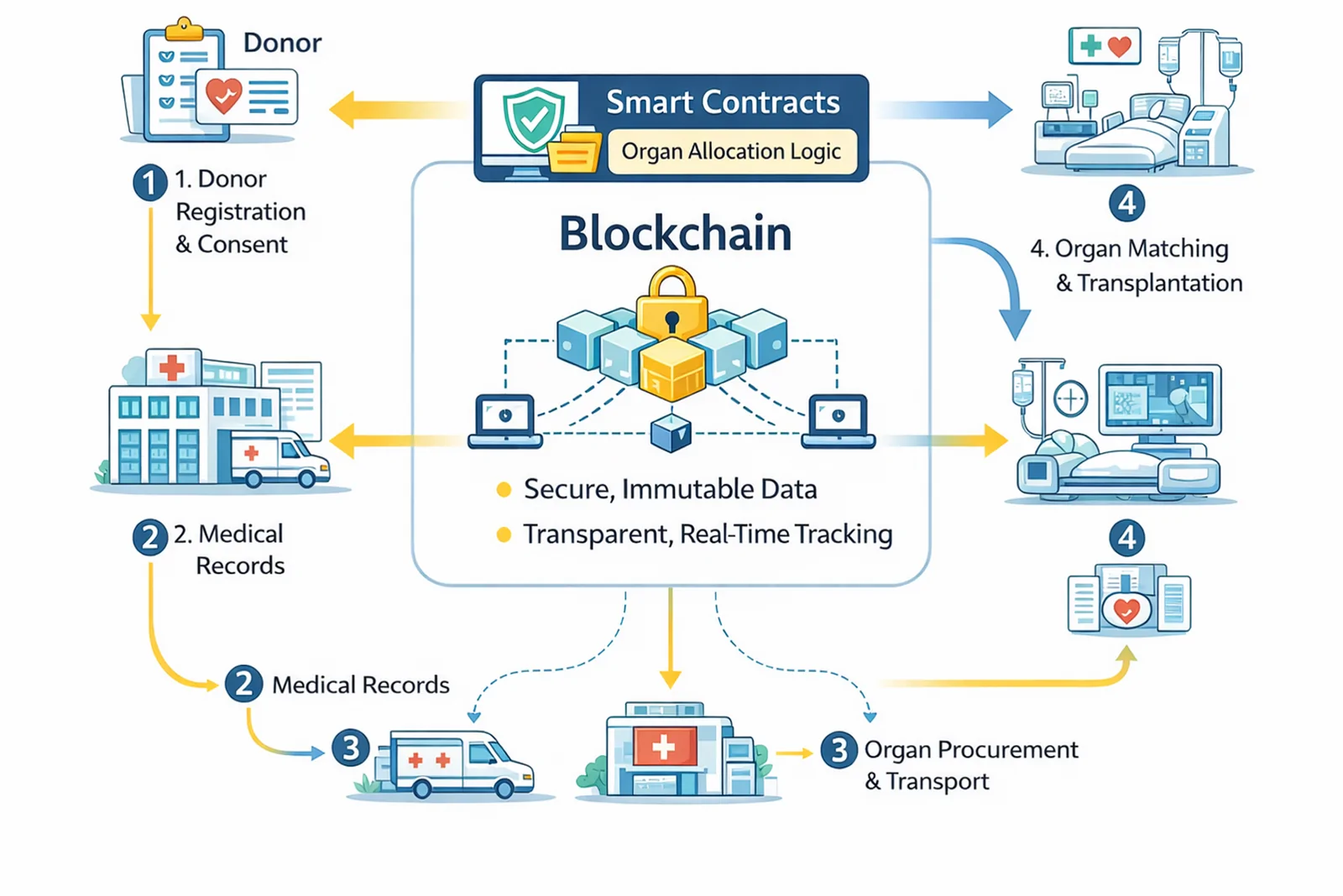
Blockchain-based system for secure and transparent organ donation and transplantation systems.

Figure 8 demonstrates that blockchain-based systems combine smart contracts, decentralized registries, and permissioned access control systems to facilitate the main steps of the transplantation pipeline, such as donor registration, medical record management, organ allocation, and transplantation. In the majority of its applications, sensitive clinical information is stored off-chain, with blockchain storing hashes, transaction history, consent history and allocation history, which ensure data privacy and integrity (47).

According to the literature, blockchain is best applied in the case where common trust among institutions is needed, but not computational efficiency. Its most effective uses are waiting list integrity, consent traceability, organ custody tracking and audit logging (48). As an example, waiting list systems that are based on blockchains can help avoid unauthorized manipulation by keeping the records of allocation decisions unchanged, whereas smart contracts can be used to enforce the allocation policies as transparent and rule-based (49).

The other significant use case is in organ logistics and traceability where blockchain with IoT technologies can be used to monitor the conditions of organ transportation and custody chains in real-time (50). This increases accountability and minimizes risks in terms of delays or mishandling during transportation.

Although these benefits exist, the present level of blockchain usage is still on the prototype or pilot level. The majority of the research is dedicated to the conceptual frameworks, simulations, or small-scale deployments based on Ethereum and Hyperledger Fabric platforms (51). Its application in real-life is restricted because of issues to do with governance, integration with healthcare systems, regulatory adherence, and identity management (52).

Moreover, although blockchain enhances transparency and auditability, it does not necessarily address the problem of fairness of allocation, shortage of organs, or complexity of clinical decisions, which are based on the policies and institutional practices. Thus, blockchain is not a solution but a coordinating infrastructure that should be considered an enabler.

As shown in Table 2, the applications of blockchain in the field of transplantation are mostly focused on improving transparency, traceability, and trust between the parties. Unlike AI, however, blockchain technologies have no direct impact on the availability of donors or how they are allocated. Most of the studies reported are conceptual frameworks or pilot implementations and very few are reported to have been deployed clinically in the real world. Further, scalability, interoperability with the current healthcare information systems, governance models and regulatory compliance issues continue to be barriers to the widespread adoption. Therefore, blockchain should be considered an infrastructure solution for secure data coordination, not as a single solution to the problems in transplantation.

**Table 2:** Applications, benefits, and limitations of blockchain in organ donation and transplantation systems.

References	Application area	Key function	Benefits	Limitations
(47)	Waiting List Management	Immutable queue records	Prevents manipulation, improves transparency	Policy-dependent fairness
(48)	Consent Management	Time-stamped consent logs	Legal traceability, accountability	Revocation complexity
(49)	Organ Allocation	Smart contract-based rules	Automated, auditable decisions	Hard to encode clinical exceptions
(50)	Logistics & Transport	Chain-of-custody tracking	Real-time monitoring, traceability	IoT integration challenges
(51)	Medical Data Sharing	Secure access control	Privacy + interoperability	Off-chain dependency
(52)	Audit & Governance	Distributed ledger logs	Transparency, trust	Governance complexity

### 5.3 Internet of medical things (IoMT) and real-time monitoring

IoMT has become one of the newest technological developments in the field of organ donation and transplantation, especially in overcoming the issue of logistics, organ preservation, and real-time coordination. IoMT is a combination of medical equipment, sensors, and communication systems that can be used to constantly monitor and exchange data during the transplantation process.

Organ preservation and transportation is one of the most important uses of IoMT in which the preservation of organs depends on the optimal conditions of the environment (53). Transport containers can also be fitted with sensors that constantly monitor the temperature, humidity, and ischemic time to ensure that organs are not exposed to unsafe conditions during transportation. The transmission of real-time data enables the healthcare provider to monitor the status of the organs and receive notifications in case of deviations, which will help to take corrective measures in time.

IoMT also improves the traceability and accountability throughout the transplantation pipeline. IoMT systems also minimize uncertainties related to logistics and enhance stakeholder coordination by offering end-to-end visibility of organ movement, i.e., between donor hospitals and transplant centers. This is especially critical in healthcare systems that are geographically dispersed, in which delays and miscommunication may have a significant effect on transplantation outcomes.

Moreover, IoMT aids in the process of making decisions based on data through the integration of real-time monitoring data with clinical information systems (54). This allows clinicians to test the quality of organs in a more precise way and make a correct decision on the appropriateness of transplantation. IoMT can also be used to enhance operational efficiency and decrease organ discard rates when it is used together with advanced analytics and predictive models.

Although these benefits exist, there are a number of obstacles that restrict the popularization of IoMT in transplantation systems. These are the issues of data security, device reliability, compatibility with the current healthcare infrastructure, and network dependence (55). The problem of ensuring the smooth integration of IoMT devices with hospital information systems and data integrity between platforms is of high importance.

As outlined in Table 3, the primary applications of IoMT in organ transplantation are the real-time monitoring, traceability, and cold chain management, whereas the key challenges are interoperability, device reliability, and data security. The use of IoMT has been shown to have a significant impact on the logistics, preservation of organs, and coordination of the transplantation process. By leveraging technologies like blockchain and AI, its integration can further optimize system performance and overcome current challenges in the organ donation and transplantation landscape.

**Table 3:** Key applications and challenges of IoMT in organ transplantation.

Application area	Key function	Benefits	Challenges
Organ transport monitoring	Sensors track temperature, humidity, ischemic time	Maintains organ viability, reduces damage risk	Sensor reliability, calibration issues
Real-time tracking	GPS-enabled tracking of organ movement	Improved coordination, reduced delays	Network dependency, connectivity issues
Cold chain Management	Continuous monitoring of preservation conditions	Minimizes organ discard, ensures quality	Integration with logistics systems
Alert systems	Automated alerts for threshold violations	Enables timely intervention	False alarms, system accuracy
Data integration	Linking IoMT data with hospital systems	Supports clinical decision-making	Interoperability challenges
Traceability & accountability	End-to-end tracking of organ handling	Enhances transparency and trust	Data security and privacy concerns

Even though IoMT has the potential to revolutionize organ preservation and logistics, it requires reliable communication infrastructure, device interoperability, and robust cybersecurity measures. Moreover, the deployment and maintenance cost of the sensor could be a constraint for its adoption in resource constrained healthcare environments. Based on this, IoMT technologies have a high degree of potential, but they require more validation and standardization for use in transplantation systems.

### 5.4 Advanced organ preservation technologies

The development of organ preservation technologies has changed the practice of transplantation to be short-term storage-oriented to graft optimization, viability testing, and long-term preservation. Whereas the most common approach to cold storage is still the use of the simplest and least expensive method of preservation, known as static cold storage (SCS), its shortcomings are becoming more apparent, especially in the case of extended criteria donors and donation after circulatory death (DCD) grafts, where quality of preservation is directly proportional to outcome (53).

**Figure 9:**
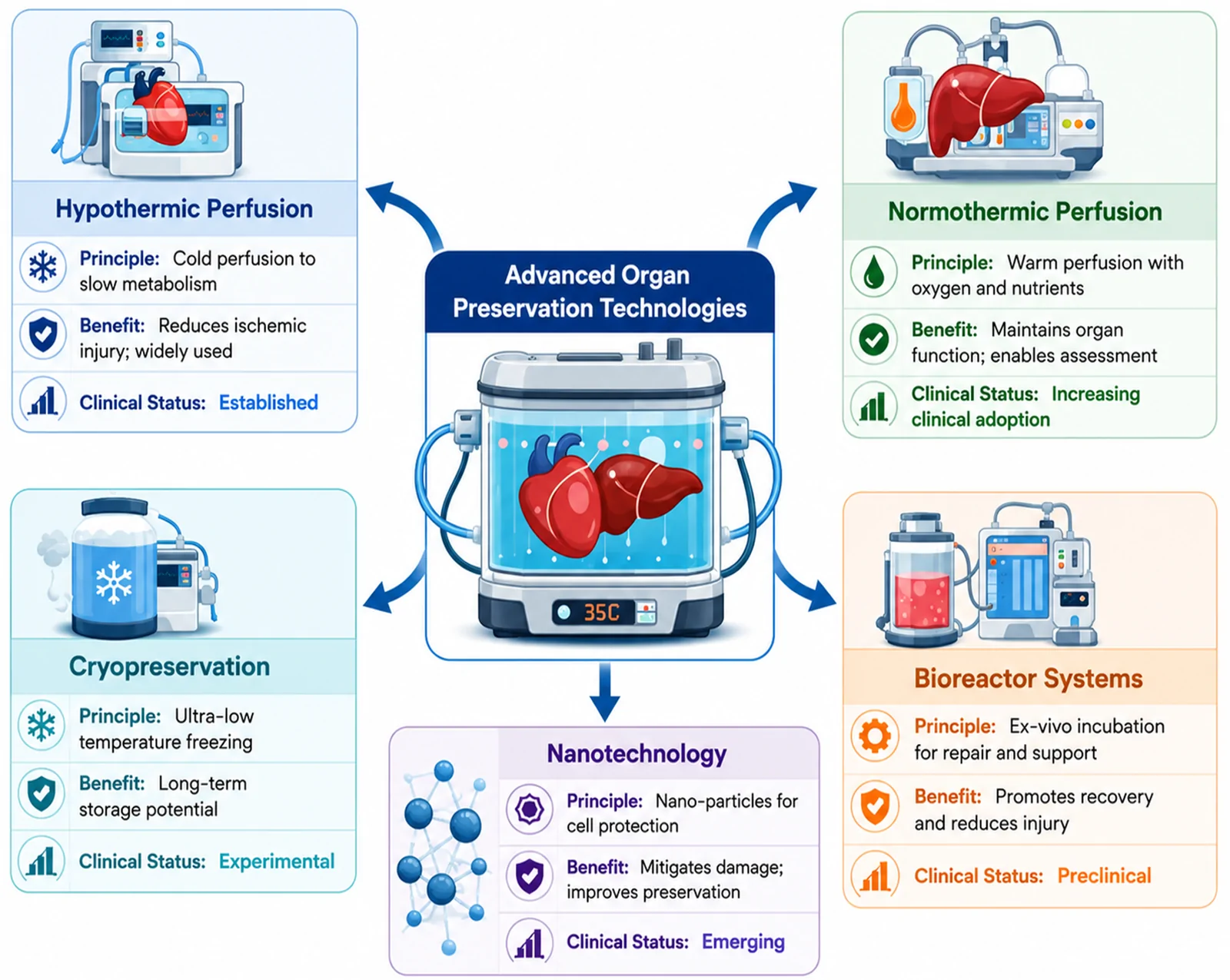
Conceptual overview of advanced organ preservation technologies in transplantation.

The greatest advancement has been made in the dynamic preservation methods, particularly machine perfusion, which makes it possible to maintain constant oxygenation and metabolic support. Hypothermic machine perfusion causes a decrease in metabolic demand with cellular integrity and is thus especially useful in high-risk grafts including kidneys and livers (54). Conversely, normothermic machine perfusion restores physiologically close conditions, and real-time functional evaluation and possible repair of organs can be performed before transplantation (55). These methods do not only enhance the survival of the grafts but also increase the donor pool since it is possible to use marginal organs. Figure 9 shows representation of the technologies of preserving advanced organs, such as hypothermic and normothermic perfusion, cryopreservation, bioreactor systems, and nanotechnology-based methods to improve the viability of organs and preserve their functionality longer.

Organ preservation strategies increasingly employ machine perfusion approaches that are tailored to specific organ types and clinical requirements Hypothermic machine perfusion (HMP) is a technique that has been employed in kidney transplantation and has shown to be beneficial in minimizing delayed graft function and enhance graft survival. Normothermic machine perfusion (NMP), on the other hand, allows organs to be perfused at near-physiologic temperatures and allows for real-time assessment of organ viability, especially for liver and heart transplantation. New technologies like supercooling, partial freezing and vitrification are being developed to significantly improve preservation times, but these are still mostly experimental and need to be clinically validated.

Preservation strategy will then be dependent on the type of organ, its ischemic tolerance and logistical considerations for transplant.

New methods, such as the sequential perfusion (cold-to-warm), are anticipated to give protection benefits of hypothermia and evaluation capabilities of normothermia (balanced approach to preservation and evaluation). All subzero preservation methods (other than perfusion) aim to extend preservation by slowing the metabolism further, such as by supercooling and partial freezing. Supercooling does not allow the formation of ice and has been found to be a useful approach in liver preservation, while partial freezing allows for controlled ice formation, but is still under investigation due to potential risks of tissue damage and complex procedures (56).

Organ nanowarming by vitrification with ice-free preservation and fast and even rewarming may enable the real long-term organ banking in the frontier. Although recent preclinical studies have demonstrated the successful transplantation in animal models, the challenges including cryoprotectant toxicity, scalability and process standardization hamper the direct clinical translation.

As summarized in Table 4, the most clinically developed technologies are machine perfusion technologies, whereas subzero and cryogenic ones are in early stages of development, but with high potential in the long-term. On the whole, the sphere is moving towards a hybrid preservation paradigm, when preservation strategies are developed and applied depending on organ type, characteristics of donors, and clinical needs instead of a universal approach.

**Table 4:** Comparative overview of advanced organ preservation technologies.

Refs.	Technology	Key principle	Advantages	Limitations	Maturity level
(53)	Static Cold Storage (SCS)	Low-temperature preservation to slow metabolism	Simple, low cost, widely used	Limited preservation time, poor support for marginal organs	Clinical standard
(54)	Hypothermic Machine Perfusion	Cold perfusion with oxygen support	Reduces ischemic injury, improves marginal graft use	Limited functional assessment	Near-clinical
(55)	Normothermic Machine Perfusion	Warm perfusion with active metabolism	Real-time assessment, organ repair capability	High cost, complex logistics	Clinically emerging
(56)	Sequential Perfusion (Cold → Warm)	Combined hypothermic and normothermic strategies	Balances protection and functional testing	Workflow complexity	Emerging
(57)	Supercooling	Subzero preservation without ice formation	Extends preservation duration	Risk of ice formation, scalability issues	Experimental
(58)	Partial Freezing	Controlled ice formation for deep metabolic arrest	Significant extension of storage time	Tissue damage risk, protocol complexity	Experimental
(59)	Vitrification & Nanowarming	Ice-free cryogenic preservation with rapid rewarming	Potential for long-term organ banking	Toxicity, scalability, high complexity	Preclinical

Advanced organ preservation technology, especially hypothermic and normothermic machine perfusion, has demonstrated great potential to enhance the utilization of organs and prolong their preservation. However, the broad adoption of these is limited by their cost, the special infrastructure needed and protocol differences between organs. Furthermore, long-term clinical and economic benefits are still conflicting, particularly with respect to pancreas and intestinal transplantation. Further studies are needed on protocol standardization, and cost-effectiveness analysis to promote wider use.

### 5.5 Big data analytics and decision support systems

Healthcare data has been an ever-increasing source of data, with the use of big data analytics and decision support systems (DSS) to organ donation and transplantation providing new opportunities to improve organ allocation, improve patient outcomes, and improve system efficiency. These technologies include sharing and access to extensive data, including electronic health records (EHRs), donor registries, transplant databases and real-time monitoring data, to support evidence-based clinical and operational decisions. Predictive modeling is one of the key applications of big data analytics, statistical and machine learning techniques that have the potential of predicting the likelihood of graft survival, patient outcomes and even the potential for complications (60). They can be used to assess the historical and real-time data to identify patterns that can help with improved donor–recipient matching and improved allocation decisions. They augment the clinical judgement with multidimensional data, which are not easily understood from the clinical point of view. The application of big data analytics is also essential in forecasting demand and supply and optimising resources. The study of trends in organ donation, waiting lists and organ transplantation activity can bring insight to healthcare systems to make better predictions of their future organ transplants and to plan policies to reduce their waiting lists and organ discard rates. This is particularly relevant to reducing the inefficiencies that exist systemically as discussed above.

Clinical decision support systems (DSS) are combinations of predictive models and real-time data, which help clinicians when they are making decisions about donors, recipients and workflow. These systems help to enhance the uniformity of clinical practice, minimize uncertainty, and ensure a more uniform process in decision making across institutions (61).

However, several issues are linked with the application of big data analytics in transplantation, including data heterogeneity, data quality issues, data interoperability and privacy issues. The issue of data from various sources and the accuracy and compliance with the regulations is a complex one (62). In addition, the complex models will be explainable to be accepted by clinicians, otherwise, the trust between medical staffs may be decreased due to the lack of explainability in the decision making process.

In general, big data analytics and decision support systems are the key facilitators of data-driven transplantation ecosystems, which enhance efficiency, transparency, and clinical outcomes. They might be integrated with the emerging technology such as artificial intelligence, blockchain, and IoMT to enhance the transplantation system's organization and intelligence.

### 5.6 Comparative analysis of emerging technologies

While there is significant potential in the use of AI, blockchain, IoMT, advanced organ preservation technologies and big data analytics to enhance transplantation systems, not all these technologies are equally mature, applicable and ready for implementation. A comparative evaluation of these technologies is needed to be able to pinpoint technologies that have immediate translation potential and those that need more research and development.

As shown in Table 5, emerging technologies differ considerably in their level of clinical maturity and implementation. Advanced organ preservation technologies and AI demonstrate the highest readiness for clinical integration due to growing validation and adoption in transplantation workflows. In contrast, blockchain and IoMT remain largely in pilot or early implementation stages because of interoperability, scalability, and regulatory challenges. Big data analytics serves as a complementary technology by supporting evidence-based decision-making but relies on standardized, high-quality datasets. These findings suggest that future transplantation systems should integrate complementary technologies rather than rely on isolated digital solutions.

**Table 5:** Comparative assessment of emerging technologies in organ transplantation.

Technology	Major applications	Advantages	Limitations	Clinical readiness	Current adoption
Artificial intelligence	Allocation, prediction	High predictive accuracy, decision support	Bias, external validation, explainability	High–Moderate	Increasing clinical adoption
Blockchain	Data sharing, traceability	Transparency, immutability	Scalability, interoperability	Low	Pilot implementations
IoMT	Monitoring, logistics	Real-time monitoring	Cybersecurity, infrastructure cost	Moderate	Emerging adoption
Advanced organ preservation	Machine perfusion	Better graft quality, longer preservation	Cost, specialized equipment	High	Increasing clinical use
Big data analytics	Decision support	Population-level insights	Data quality, governance	Moderate	Growing use in transplant registries

Therefore, a multi-disciplinary approach including technological innovations, clinical validation, regulatory and implementation strategies, will be needed for successful implementation of transplantation systems.

Although new technologies hold great promise, they are not yet into the mainstream of transplants. Algorithmic bias and data drift are challenging to address in AI-based systems because they need a lot of external validation and monitoring to prevent these issues. Likewise, blockchain and IoMT platforms are both beset with interoperability, scalability and cybersecurity issues which could impede widespread adoption. Additionally, digital infrastructures must be, to some degree, financially expensive and technically complex, and in low-resource communities, may lack the financial resources and technical skills needed to implement them.

In addition to technical issues, regulatory clearance, clinician receptivity and strong governance structures for sharing and ethical oversight of data must be developed for successful implementation. Therefore, the next steps should be directed towards technology innovation and implementation strategies that will enable the safe, equitable and clinically sustainable implementation of such technologies within transplantation systems.

Overall, the reviewed technologies demonstrate different levels of clinical maturity and practical applicability. Artificial intelligence and advanced organ preservation technologies currently exhibit the highest translational potential owing to increasing clinical validation and integration into transplantation workflows. In contrast, blockchain and IoMT remain largely in the early stages of implementation, with their widespread adoption limited by interoperability, regulatory, scalability, and cybersecurity challenges. Big data analytics complements these technologies by enabling evidence-based decision-making but depends on standardized, high-quality datasets and robust governance frameworks. Rather than functioning as standalone solutions, these technologies are expected to provide the greatest clinical benefit when deployed as integrated components of a digital transplantation ecosystem that supports secure data exchange, intelligent decision support, real-time monitoring, and optimized organ preservation.

## 6 Future directions and research opportunities in organ donation and transplantation

The main challenge in the future for organ donation and transplantation will not be technological advances, but the successful integration of current technology into the new, seamless and scalable systems. Although, all three technologies—AI, blockchain and the Internet of Medical Things (IoMT)—have shown potentials, their use has only resulted in marginal benefits. The second step is the construction of the interconnected transplant systems, where the intelligent forecasting, safe data exchange and real-time monitoring leads the way. This integration will certainly make the matching of the donor and recipient much easier, simplify the whole process and facilitate coordination.

Today, however, the transplantation systems are highly fragmented, and the data is spread out across hospitals and regional transplantation registries, as well as national databases (which may not interoperate). This fragmentation makes it difficult to make decisions in time and wider use of data-driven insights. There are several recommendations for future work, specifically to standardize data models, enhance interoperability and create secure data-sharing protocols (64). The ease of use and the level of privacy protection, especially in relation to cross-border transfers of data, must be taken into account in the solutions. While technical issues cannot be ignored, so must not be ethical and regulatory issues. AI tools for organ allocation, however, raise concerns regarding bias, fairness, and explainability while blockchain systems raise governance and accountability concerns. Hence, robust systems of validation, ethical guidelines and transparent laws and regulations are needed for the responsible use of these technologies. Even if they are good ideas, solutions can fail to be accepted in the clinical environment if they are not there.

The organ preservation and bioengineering is the next promising development. Machine perfusion and other methods are improving times and viability of the grafts with vitrification. In the meanwhile, tissue engineering and bioartificial organs may contribute to reduce the donor requirement. These methods, however, have yet to be clinically validated and cost-effectiveness and scalability of these methods needs to be assessed more widely (1). Equally important is to tackle inequity in access to transplantation. High income and low and middle income areas have a huge infrastructure gap, lack of awareness and policies. Capacity building, international cooperation and cost effective and scalable solutions to resource-poor environments are needed to address these gaps. Increasing public participation and improving the deceased donor programs will also contribute to improving the supply of organs.

Lastly, the field is shifting to a more data-driven and patient-centred approach. These AI-powered tools, along with real-time monitoring, predictive analytics, and tailored care, can further improve patient outcomes and overall transplant experiences (65). However, the success of this vision will depend on the match between the technological innovation and clinical workflows, and on the policy support. In this respect, the future of transplantation is not solely about new technologies but about digital innovation and the effective combination of clinical skills and good governance that will drive efficient, equitable and patient-centred transplantation systems.

Table 6 outlines key research areas that need to be further explored to develop integrated digital transplantation ecosystems with the incorporation of artificial intelligence, secure data-sharing platforms, real-time monitoring technologies, and advanced preservation strategies. There is a need to focus on explainable and equitable AI frameworks, establishing standard data infrastructures and identifying low-cost solutions that can be adopted in various healthcare settings including in low-resource areas.

**Table 6:** Future research directions in organ donation and transplantation.

Domain	Key challenges	Emerging solutions	Future research opportunities
Technology integration	Isolated implementation of AI, blockchain, IoMT	Integrated digital transplantation ecosystems	Development of unified architectures for real-time coordination and decision support
Data infrastructure	Fragmented registries and poor interoperability	Standardized data formats and interoperable platforms	Global frameworks for secure, cross-institutional data exchange
Ethics & governance	Bias in AI models, lack of transparency	Explainable AI, regulatory guidelines	Fair allocation algorithms, accountability mechanisms, and ethical validation models
Organ preservation	Limited preservation time and organ viability	Machine perfusion, supercooling, vitrification	Scalable and cost-effective preservation technologies with clinical validation
Bioengineering	Dependence on donor organs	Tissue engineering, bioartificial organs	Translational research for clinical adoption and long-term functionality
Global access	Regional disparities in transplantation rates	Capacity building initiatives, policy reforms	Affordable and scalable transplantation models for low-resource settings
Patient-centric systems	Limited personalization in treatment	Predictive analytics and precision medicine	Adaptive, outcome-driven transplantation frameworks

## 7 Conclusion

Organ donation and transplantation have developed to a high level, but the main obstacles, including the ineffective use of the donors, geographical disparities, logistics, socio economic factor and data fragmentation are still limiting its effectiveness. The problems show the challenges of the transplantation systems and are not individual failures. New technologies, including artificial intelligence, blockchain and IoMT will help to improve the allocation, transparency and monitoring. However, what really counts is if they are successful in integrating into clinical practice and supported by appropriate regulatory and ethical standards. The advancement will not be solely based on technological innovation but also on policy support, capacity building as well as international collaboration. Finally, while the efforts are encouraging, technology, policy, and practice changes will have to continue to be implemented on a multi-faceted basis over time, in order to make transplantation system truly efficient and equitable (63).
